# Beneath the canopy, beneath the ground: how surface microhabitats shape cave communities

**DOI:** 10.7717/peerj.20593

**Published:** 2026-01-09

**Authors:** Marcus P. A. Oliveira, Rodrigo L. Ferreira

**Affiliations:** 1 BioEspeleo Consultoria Ambiental, Lavras, MG, Brazil; 2 Center of Studies in Subterranean Biology, Ecology and Conservation Departament, Universidade Federal de Lavras, Lavras, MG, Brazil

**Keywords:** Cave conservation, Invertebrates, Amazon biodiversity, Leaf litter fauna, Iron ore

## Abstract

Ecosystem dynamics are shaped by spatial and temporal fluctuations in resource availability and species composition across the landscape. Caves exemplify this interconnectedness, as they rely on allochthonous inputs and surface climatic conditions, fostering communities composed of viable populations from both surface and subterranean habitats. Consequently, effective management and conservation of subterranean environments require the protection of adjacent surface ecosystems, a particularly urgent need in the Amazonian ferruginous hills, where mineral exploitation poses a significant threat. In this context, we investigated how vegetation cover and environmental variables influence the distribution of surface and subterranean fauna, and which factors should guide conservation strategies. We sampled six caves and ten epigean transects around each, encompassing both ombrophilous forest and canga (savanna over ferruginous crusts). Environmental structure variables were recorded for all transects. Significant differences were detected between forest and canga in terms of vegetation structure, microclimate, species richness, and community composition. Species most similar to those found in caves were associated with forest leaf litter, particularly in transects closest to cave entrances. Leaf litter depth emerged as a key factor facilitating faunal overlap between surface and subterranean habitats, likely by providing microhabitats with stable temperature and humidity. In contrast, faunal similarity in canga occurred only under specific conditions, namely, milder temperatures, deeper litter layers, and increased canopy cover. Our findings demonstrate that cave-dwelling species in Amazonian ferruginous systems also inhabit adjacent forest environments, which should be prioritized for conservation.

## Introduction

Interactions between adjacent ecosystems and their influence on ecological processes are central themes in landscape ecology and conservation (Clark, 2010). An integrated understanding of ecosystems and landscapes is essential for elucidating the spatial and temporal dynamics of resource and species flows, including water, nutrients, and organisms (Costanza et al., 2002). This perspective is particularly important for caves—subterranean karst systems embedded within a wide range of land-use contexts across the globe (Williams, 2008). The deepest cave zones display distinctive characteristics compared to surface ecosystems, such as permanent darkness, stable temperatures, and high humidity, often supporting obligate troglobitic species that are strictly adapted to these environments (Romero, 2009). Their ecological integrity depends on the balance among abiotic (*e.g*., rock matrix, water, soil), biotic (*e.g*., fauna, trophic inputs), and atmospheric (*e.g*., temperature, humidity) components (Howarth & Wynne, 2022; Elez et al., 2013). However, due to their geological significance and the high economic value of associated minerals, cave landscapes are frequently subject to urban, agricultural, and industrial pressures (Ferreira, Oliveira & Silva, 2018; Jaffé et al., 2018).

The absence of light in deeper reaches of caves precludes primary production through photosynthesis, making these ecosystems heavily reliant on allochthonous inputs. Landscape characteristics also influence subterranean climate, with cave temperatures at depth generally reflecting the annual mean of surface conditions (Badino, 2010; Mammola et al., 2019). Consequently, subterranean communities are shaped by interactions between epigean and hypogean systems, particularly in tropical regions where many species maintain viable populations across both habitats (*i.e*., troglophiles) (Lunghi, Manenti & Ficetola, 2017; Mammola & Isaia, 2018). Therefore, conserving subterranean ecosystems requires protection not only of the caves themselves but also the surrounding landscapes that influence their ecological dynamics (Xiao & Weng, 2007; Elez et al., 2013). Nonetheless, many caves remain highly vulnerable due to insufficient protective measures, especially in the ferruginous highlands of the Brazilian Amazon, where mining, livestock grazing, and agricultural expansion threaten cave habitats (Oliveira & Ferreira, 2024; Ferreira, Oliveira & Silva, 2018; Jaffé et al., 2018).

**Figure 1:**
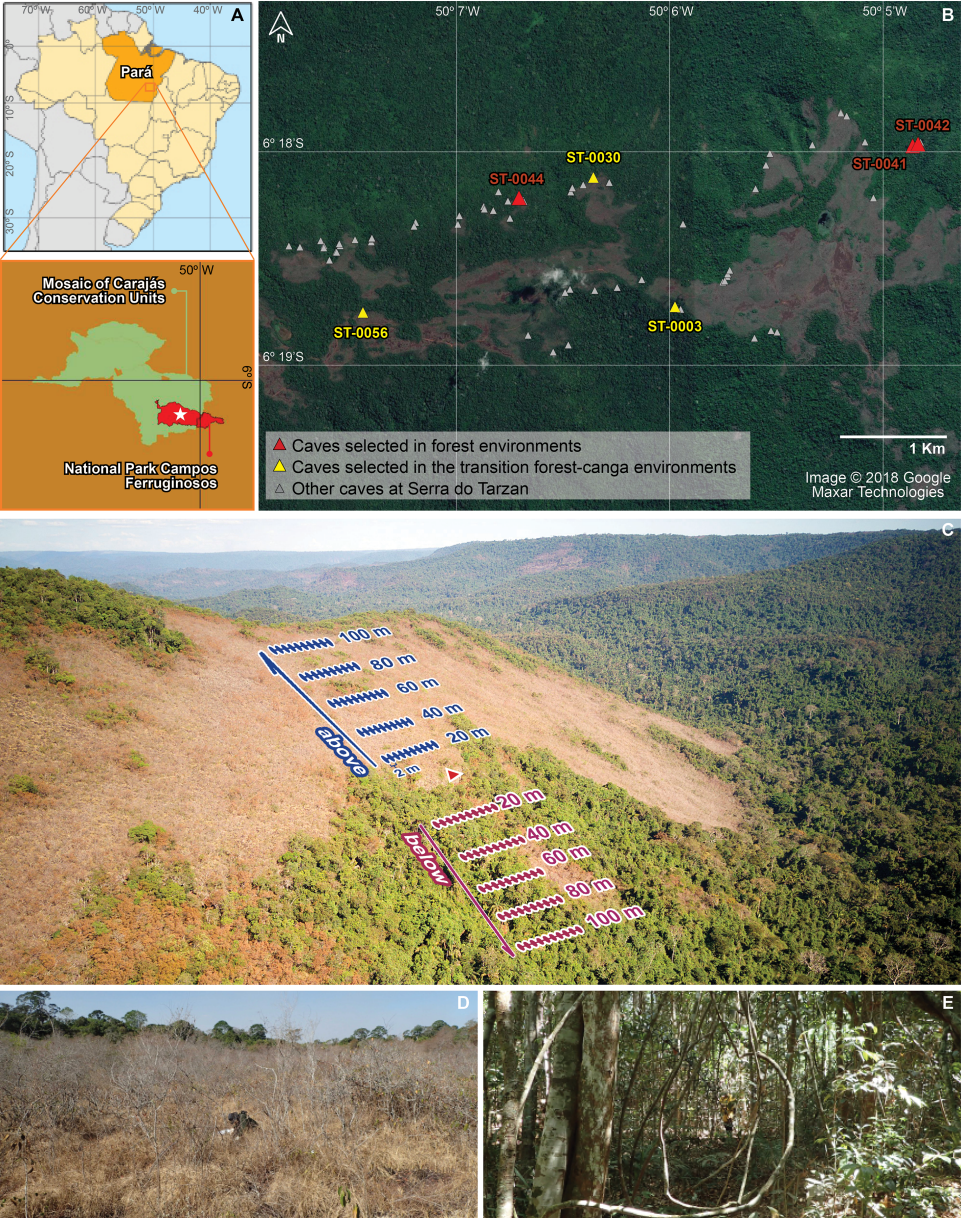
Study area location and sampling design. (A) The Campos Ferruginosos National Park is located in the southeastern Amazon, in the state of Pará, Brazil. Serra do Tarzan (white star) is one of the geomorphological units within this protected area. (B) Among the caves found in Serra do Tarzan, six were selected based on the predominant surrounding vegetation: three located in ombrophilous forest (red triangles) and three at the interface between forest and canga (yellow symbols). ST-0041 cave (6°18′49.49″S, 50° 5′36.46″O), ST-0042 cave (6°18′49.31″S, 50° 5′36.02″O) and ST-0044 cave (6°19′5.93″S, 50° 7′37.84″O) and three in the intersection between forest and canga: ST-0003 cave (6°19′39.87″S, 50° 6′48.48″O), ST-0030 cave (6°19′0.69″S, 50° 7′15.41″O) and ST-0056 cave (6°19′40.97″S, 50° 8′25.40″O). (C) Sampling design implemented in the Amazonian ferruginous hills. Five equidistant transects (20 m apart) were established above and below the cave entrances. Each transect was subdivided into ten equidistant sampling points (2 m apart). (D) Canga area sampled above the entrance of cave ST-0056. (E) Sampling below the same cave occurred in forested transects. Map data © 2018 Google/Maxar Technologies.

Ferruginous caves in the Amazon are generally small (averaging 30 m in length), shallow, and composed of networks of interconnected voids that facilitate faunal exchange with surrounding epigean environments (Piló, Coelho & Reino, 2015; Ferreira, Oliveira & Silva, 2018). These caves are typically found along plateau escarpments and colluvial slopes—landforms susceptible to erosion from rainfall (Piló, Coelho & Reino, 2015). As a result, they often coincide with ecotones between contrasting vegetation types: rupestrian/shrubby savannas over ferruginous crusts (canga) and forest formations associated with perennial or intermittent streams (Mota et al., 2015) (see Fig. 1). The stark differences between these vegetation types raise important questions about the origin and composition of cave-dwelling communities.

In this study, we investigated how vegetation cover and landscape-scale environmental factors influence the distribution of epigean and hypogean species and identified the variables most critical for cave conservation. Specifically, we aimed to: (i) evaluate whether cave communities resemble those of adjacent epigean phytophysiognomies; (ii) assess whether the proximity of surface habitats to caves influences the presence of surface species within caves; (iii) identify which environmental attributes of forest and canga landscapes best explain the similarity between surface and subterranean faunas; and (iv) determine threshold values for key predictors that promote the ecological connectivity required for cave conservation.

We hypothesized that cave faunas are equally derived from both adjacent forest and canga environments, primarily influenced by their spatial proximity to caves. Additionally, we predicted that while vegetation type would influence species composition, it would not affect the number of taxa shared between surface and subterranean habitats. In epigean environments, we expected species occurrence to be driven by microhabitat conditions, particularly temperature and relative humidity, regardless of vegetation type.

## Methods

### Study area

We conducted our study in Serra do Tarzan (6°19′51.16″S, 50°7′58.15″W), a geomorphological unit located within Campos Ferruginosos National Park, southeastern Pará State, Brazil (Fig. 1). This national park is part of the Carajás Mosaic—a cluster of protected areas surrounding the Carajás Mineral Province, a globally significant region for iron, nickel, copper, and gold extraction (Piló, Coelho & Reino, 2015). Over 1,000 caves have been documented in the region, with at least 305 located within the national park (Piló, Coelho & Reino, 2015).

The regional climate is equatorial, varying with elevation: below 350 m a.s.l., it is classified as equatorial continental (mean annual temperature ~25 °C; annual rainfall 1,900–2,000 mm), while above 700 m a.s.l., it becomes mesothermal equatorial (mean annual temperature ~23 °C; rainfall up to 2,400 mm) (Piló, Coelho & Reino, 2015). Rainfall is highly seasonal, with a dry season from May to September (~10–90 mm/month) and a wet season from October to April (~160–340 mm/month) (Sahoo et al., 2016).

#### Sampling design

We sampled six caves from July to August 2016: three located in ombrophilous forest (ST-0041, ST-0042, ST-0044) and three in ecotonal zones between forest and canga (ST-0003, ST-0030, ST-0056) (Fig. 1). At each cave, we established ten 20 m-long transects extending from the entrance—five uphill and five downhill. Each transect was subdivided into ten 1 × 1 m sampling units spaced every 2 m.

### Environmental variables

We measured six environmental variables per transect: temperature (°C), relative humidity (% RH), altitude (m a.s.l.), canopy openness (%), and leaf litter depth (mean and standard deviation, in cm). Temperature and relative humidity were recorded at the center of each transect at ground level, following the protocol recommended by Souza-Silva et al. (2021), Reis-Venâncio et al. (2022), and Vaz et al. (2025), using a digital thermohygrometer (Incoterm 7666.02.0.00; ±0.5 °C, ±2.5% RH). Measurements were taken between 5:00 and 6:00 p.m. after a 15-min stabilization period. Altitude was measured using a Garmin 62csx GPS.

To assess canopy openness, we captured hemispherical photographs at ground level using a Sony Alpha NEX-3 camera with an 8 mm fisheye lens, mounted on a fixed platform. Images were analyzed in Gap Light Analyzer 2.0 (Frazer, Canham & Lertzman, 1999). Leaf litter depth was measured at each sampling point using a millimeter ruler, and both mean and standard deviation were calculated per transect to capture overall accumulation and spatial heterogeneity (Roeder et al., 2022; Barrientos, 2012; Didham, 1998).

### Fauna sampling and identification

We employed direct intuitive searches to sample invertebrates both within the caves and in the sampling units along the surrounding transects (Wynne et al., 2019). Substrates such as leaf litter, logs, and rocks were carefully examined using trays, tweezers, and brushes. In the areas surrounding each cave, invertebrates were sampled across the 100 sampling units established along transects positioned above and below the entrances (Fig. 1C). Inside the caves, the entire internal area was surveyed due to the spatial constraints and oligotrophic conditions characteristic of these environments, which generally sustain low faunal abundance, with individuals typically aggregated in resource-rich microhabitats. Sampling effort under both conditions (inside caves and along external transects) was standardized at 6 min/m² per collector. Permission was given by Instituto Chico Mendes de Conservação da Biodiversidade (ICMBio), N° 83/2016, regarding the protocol N° 36/2016.

All specimens were preserved in 100% ethanol and identified to the lowest possible taxonomic level by taxonomic experts (see Acknowledgments). When species-level identification was not possible, we grouped specimens into morphospecies—a valid approach for biodiversity and conservation studies (Oliver & Beattie, 1996; Pellegrini et al., 2016; Oliveira et al., 2019). We compared epigean and hypogean specimens to determine shared taxa. Immature individuals were excluded due to the absence of diagnostic characters.

Voucher specimens were deposited in the following collections: Reference Collection of Soil Fauna of Paraíba State University (Collembola), Special Laboratory of Zoological Collections, Instituto Butantan (Araneae, Diplopoda, Chilopoda), and the Subterranean Invertebrate Collection of the Federal University of Lavras (other taxa).

### Data analysis

#### Vegetation type characterization

We used Generalized Linear Models (GLMs) to test for differences in environmental variables between forest and canga transects. Variables included temperature, relative humidity, altitude, canopy openness, and leaf litter depth (mean and standard deviation). We applied negative binomial distributions with log link functions when data exhibited overdispersion relative to Gaussian models, as indicated by the Shapiro–Wilk test, which identified deviations from normality requiring a nonparametric analytical framework.

#### Biodiversity patterns across habitats

Species richness was calculated as the number of distinct taxa per transect or cave. To assess faunal similarity, we applied the Bray-Curtis index using standardized and log-transformed abundance data [log(x + 1)]. We then calculated pairwise similarity values between each transect and its nearest cave.

We used GLMs to test whether richness or similarity varied with vegetation type, distance to the nearest cave, or sampling substrate (leaf litter *vs*. under rocks or fallen tree trunks). The normality test indicated the need for a nonparametric analytical framework; therefore, negative binomial distributions were applied to account for count data overdispersion, and model residuals were examined to assess goodness of fit.

#### Community composition and environmental drivers

We performed a non-metric multidimensional scaling (nMDS) to visualize compositional differences between forest and canga transects. Subsequently, a permutational multivariate analysis of variance (PERMANOVA) was applied to test whether species composition differed significantly among predictor categories, using multiple pairwise permutations to obtain F_PERMANOVA_ values and significance levels (*p* < 0.05).

To identify predictors of faunal similarity between surface and subterranean habitats, we built separate GLMs for each vegetation type. Predictor variables included temperature, relative humidity, altitude, canopy openness, and litter depth (mean and standard deviation). Prior to modeling, we screened for multicollinearity using Spearman correlations (*rho* > 0.70, *p* < 0.05). Non-significant predictors were sequentially removed from full models based on Wald test *p*-values (*p* < 0.05). Model selection followed Akaike’s Information Criterion corrected for small samples (AICc), retaining models with ΔAICc < 2 (Burnham, Anderson & Huyvaert, 2011).

We averaged coefficients across top models to identify predictors with high uncertainty (*i.e*., wide standard deviations; Vierling et al., 2013) and conducted hierarchical partitioning to estimate each variable’s independent contribution (MacNally, 2000; Murray & Conner, 2009).

#### Breakpoint analysis

To detect potential thresholds in significant predictors, we applied piecewise regression and compared linear *vs*. segmented models based on AICc (Ochoa-Quintero et al., 2015; Magnago et al., 2015). When segmented models were preferred, we reported ΔAICc and breakpoint values.

All GLMs were performed in R 4.0.2 (R Core Team, 2024) using the *lme4* and *segmented* packages. We used PRIMER & PERMANOVA 6.0 for Bray-Curtis similarity calculations, nMDS and PERMANOVA analysis (Anderson, Gorley & Clarke, 2008).

## Results

### Characterization of vegetation types

**Figure 2:**
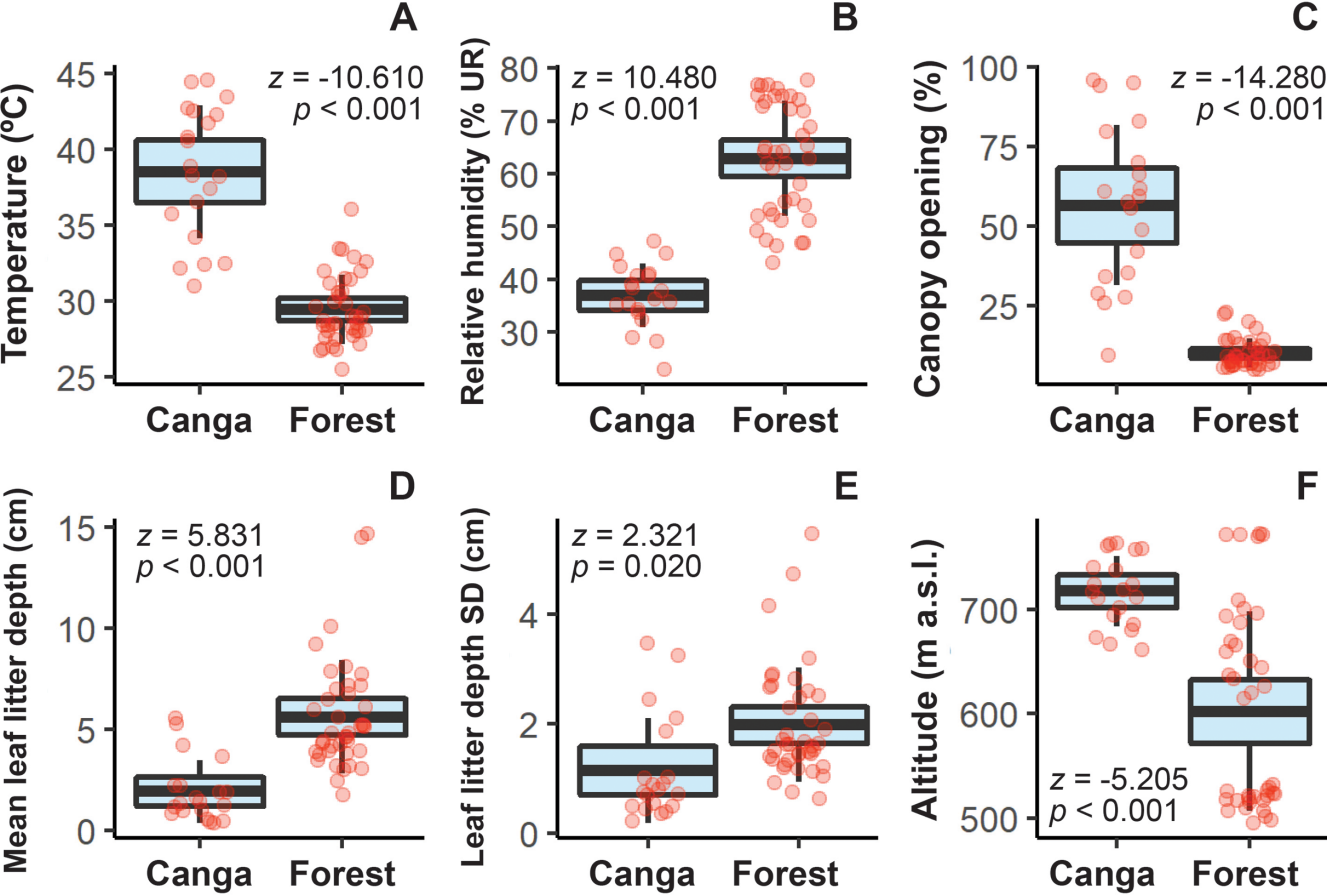
Comparison of environmental structure between forest and canga environments surrounding the caves. Response variables are followed by their respective z- and *p*-values. In the boxplots, blue areas indicate the 95% confidence interval around the observed mean (black central line), while bars represent standard deviation. Light red dots denote individual sampling units (transects). Complete statistical results are presented in Table S1.

Although forests and canga occur in adjacent areas, we observed significantly different environmental conditions between these vegetation types surrounding the caves (Figs. 2A–2E; Table 1). Forests were located at lower elevations than canga and were characterized by milder temperatures, higher relative humidity, reduced light incidence, and both greater depth and spatial variability of the leaf litter layer (Figs. 2A–2F, Table S1).

**Table 1:** Characterization of sampled transects in epigean environment according to the distance to the cavity and vegetation type (forest or canga). Similarity with cave fauna expresses values obtained by the Bray-Curtis index (BC index) assessed between each pair of transect and the closest cave.

Cave	Epigean transect data											
	Distance from the cave (m)	Vegetation type		Similarity with cave fauna (BC Index)			Altitude (m. a.s.l.)	Temperature (°C)	Humidity (% UR)	Mean Leaf litter depth (cm)	Leaf litter depth SD (cm)	Canopy opening (%)
				Fauna under rocks or fallen tree trunks	Leaf litter fauna	All transect fauna						
ST-0003	20	Canga		–	2.070	2.042	710.844	32.200	45.000	5.550	3.242	9.470
	40	Canga		0.000	7.805	8.758	717.210	32.400	47.000	4.200	1.868	28.730
	60	Canga		–	0.165	0.111	724.413	38.300	34.000	2.200	2.098	33.950
	80	Canga		0.000	1.663	0.124	737.356	36.500	38.000	1.150	0.707	48.790
	100	Canga		0.324	1.310	0.448	740.564	38.900	35.000	1.450	1.004	66.270
	20	Forest		1.552	8.562	5.157	709.102	32.000	47.000	6.800	1.634	14.020
	40	Forest		9.924	3.260	6.293	700.895	27.500	58.000	5.200	1.206	11.240
	60	Forest		2.455	4.111	4.935	696.365	27.700	55.000	4.400	1.342	7.900
	80	Forest		–	1.447	0.414	693.360	28.100	55.000	6.100	1.517	13.540
	100	Forest		–	5.773	5.332	687.270	28.100	51.000	6.000	1.625	8.860
ST-0030	20	Canga		1.121	1.215	0.075	661.000	40.600	41.000	1.200	0.489	82.920
	40	Canga		0.000	0.000	0.000	667.000	42.500	38.000	0.450	0.350	79.690
	60	Canga		0.000	0.338	0.000	673.000	42.700	39.000	0.400	0.224	69.860
	80	Canga		1.825	0.426	0.081	680.000	42.300	36.000	0.550	0.487	60.990
	100	Canga		0.668	0.000	0.723	686.000	41.800	41.000	2.250	2.439	55.440
	20	Forest		–	7.112	3.195	637.000	30.600	67.000	9.200	4.733	4.890
	40	Forest		0.000	8.880	7.320	634.000	33.500	61.000	3.000	0.757	12.780
	60	Forest		–	14.407	10.166	626.000	29.000	69.000	3.450	1.900	6.240
	80	Forest		–	7.442	6.953	620.000	31.200	62.000	7.200	2.821	7.270
	100	Forest		0.000	3.075	0.529	615.000	30.700	64.000	5.150	1.449	6.540
ST-0041	20	Forest		–	1.234	1.844	521.000	26.800	77.000	5.200	1.438	14.100
	40	Forest		–	1.292	1.793	524.000	26.900	77.000	2.450	1.393	11.460
	60	Forest		0.000	0.828	0.982	527.000	26.800	77.000	3.200	1.789	10.250
	80	Forest		0.000	6.237	7.570	529.000	27.200	76.000	3.050	1.503	7.480
	100	Forest		–	0.597	0.000	532.000	27.000	75.000	4.800	2.914	6.520
	20	Forest		–	1.710	2.001	517.000	28.100	78.000	4.150	1.677	6.510
	40	Forest		0.000	0.702	0.000	519.000	28.400	75.000	3.900	1.583	7.660
	60	Forest		0.000	6.809	5.198	517.000	28.400	74.000	3.900	1.325	22.770
	80	Forest		–	1.191	0.955	520.000	29.000	72.000	7.200	2.881	10.660
	100	Forest		–	1.031	1.143	518.000	28.500	75.000	4.600	1.170	5.530
ST-0042	20	Forest		–	0.612	0.901	507.304	28.700	62.000	5.100	2.700	17.810
	40	Forest		4.448	3.861	5.031	515.272	28.200	64.000	7.850	1.202	9.750
	60	Forest		–	0.450	0.181	521.000	28.900	63.000	7.000	2.676	9.370
	80	Forest		–	0.293	0.464	523.000	29.300	65.000	6.500	1.715	14.950
	100	Forest		0.000	4.196	5.087	526.000	29.800	65.000	3.150	1.044	12.280
	20	Forest		–	2.563	3.493	510.000	27.600	75.000	3.900	2.070	5.600
	40	Forest		0.000	1.371	1.385	507.000	32.900	64.000	5.600	2.512	8.360
	60	Forest		–	0.441	0.453	502.000	28.000	73.000	8.100	2.300	6.440
	80	Forest		–	4.002	5.836	498.000	28.500	74.000	3.800	1.495	22.470
	100	Forest		–	0.294	0.166	495.000	28.500	72.000	1.750	0.637	19.950
ST-0044	20	Canga		0.000	0.000	0.000	695.000	44.500	34.000	0.950	0.447	57.530
	40	Canga		0.000	0.040	0.000	702.000	43.500	32.000	1.900	0.895	61.870
	60	Canga		0.293	0.000	0.000	712.000	44.600	29.000	1.000	0.714	41.970
	80	Canga		0.290	0.060	0.000	719.000	34.200	41.000	5.300	3.477	35.320
	100	Canga		0.159	0.068	0.185	724.000	31.000	45.000	3.650	0.794	25.760
	20	Forest		–	2.943	0.138	670.000	32.000	47.000	14.500	4.150	14.240
	40	Forest		–	1.218	0.950	666.000	32.600	51.000	7.700	3.199	7.170
	60	Forest		0.000	0.360	0.141	660.000	31.500	49.000	10.100	5.478	6.680
	80	Forest		0.000	0.542	0.000	651.000	31.400	53.000	14.700	2.480	4.910
	100	Forest		0.000	1.321	0.000	644.000	36.100	46.000	4.300	1.446	9.050
ST-0056	20	Canga		0.323	0.114	0.000	764.000	32.500	42.000	1.900	1.019	27.470
	40	Canga		1.626	1.537	1.603	763.000	38.200	28.000	0.800	0.548	59.420
	60	Canga		0.000	–	0.000	761.000	35.800	36.000	1.600	0.747	95.930
	80	Canga		0.000	0.013	0.000	759.000	40.800	23.000	0.350	0.387	94.920
	100	Canga		0.000	0.000	0.000	758.000	37.400	35.000	1.350	0.907	94.210
	20	Forest		–	0.913	1.033	770.000	30.300	52.000	4.800	1.621	7.150
	40	Forest		–	1.119	0.652	772.000	25.500	47.000	4.650	1.252	9.000
	60	Forest		0.000	0.934	0.603	773.000	29.600	52.000	3.750	0.925	8.770
	80	Forest		–	0.641	0.082	772.000	29.900	54.000	4.400	1.121	6.960
	100	Forest		0.000	0.646	0.081	772.000	33.400	43.000	4.250	2.599	6.100

**Note:**

SD, standard deviation.

### Diversity assessment between epigean transects and caves

**Figure 3:**
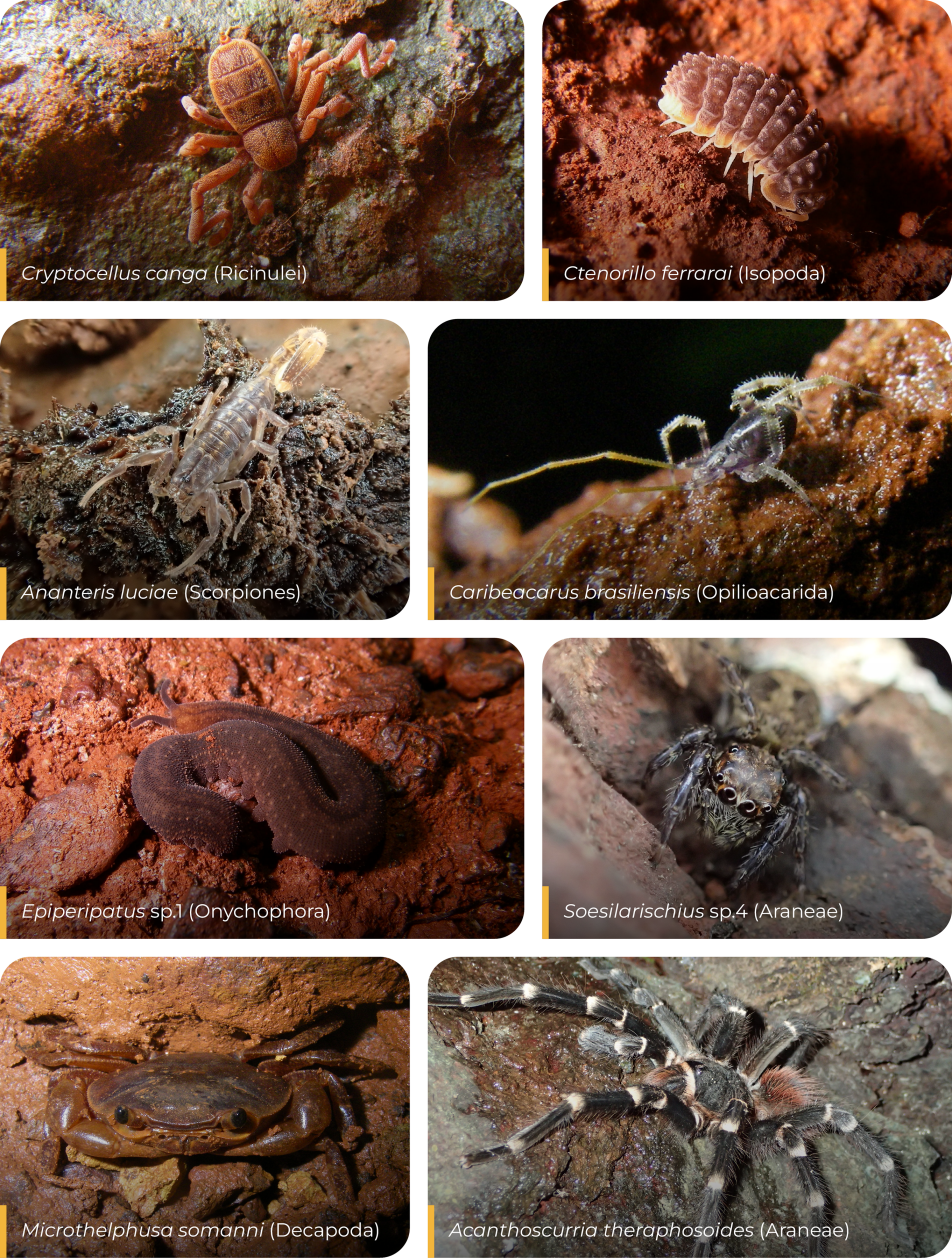
Examples of fauna sampled in Amazonian caves and their surrounding transects. Most species were recorded exclusively within caves during the sampling period. However, the mite of the genus *Caribeacarus* was found both inside the caves and in the adjacent forest and canga areas, whereas the jumping spider *Soesilarischius* occurred exclusively in forest habitats.

**Figure 4:**
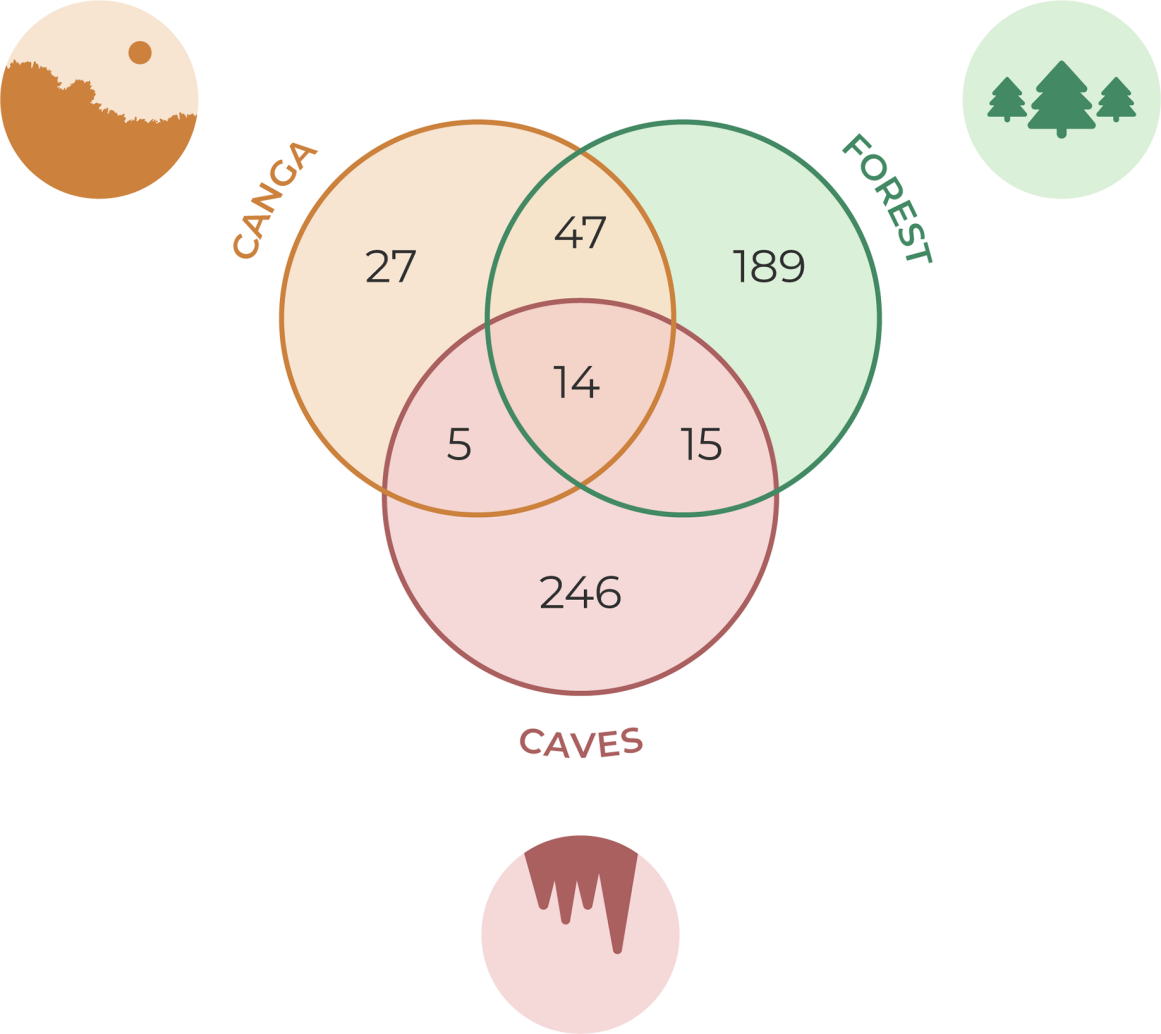
Number of species recorded in each environment and across habitats. Most species were found in caves and forest areas, while few were exclusive to the canga environment.

**Figure 5:**
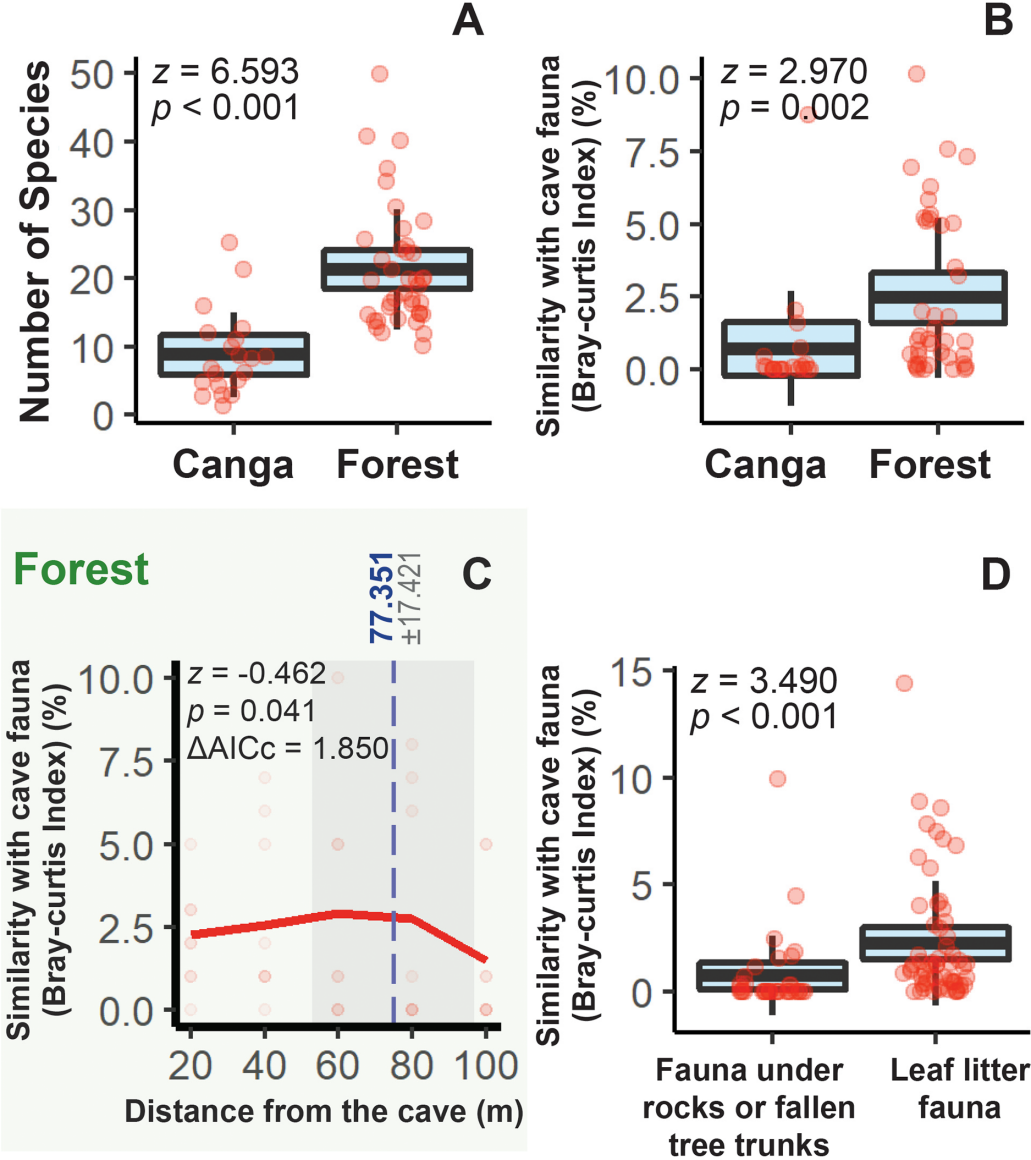
Faunal parameters related to the interface between surface and cave ecosystems. (A) Species richness according to the vegetation type in which sampled transects were inserted. (B) Similarity between cave and epigean fauna sampled in forest and canga. (C) Relationship between the species composition similarity and distance between forest transects and the cavity. (D) Similarity between cave and epigran fauna according to the substrate type in which specimens were found. Response variables are shown followed by the *z*-values and *p*-values. In boxplots, blue areas refer to the confidence interval (95%) around the observed mean (central line in black) and bars represent the standard deviation. Light red dots represent the sampling units (transects). In the piecewise regression analysis, the AICc variation relative to the linear model is reported. The dashed vertical line (in blue) indicates the significant breakpoint (*p* < 0.05) and the grey area represents the standard error.

**Figure 6:**
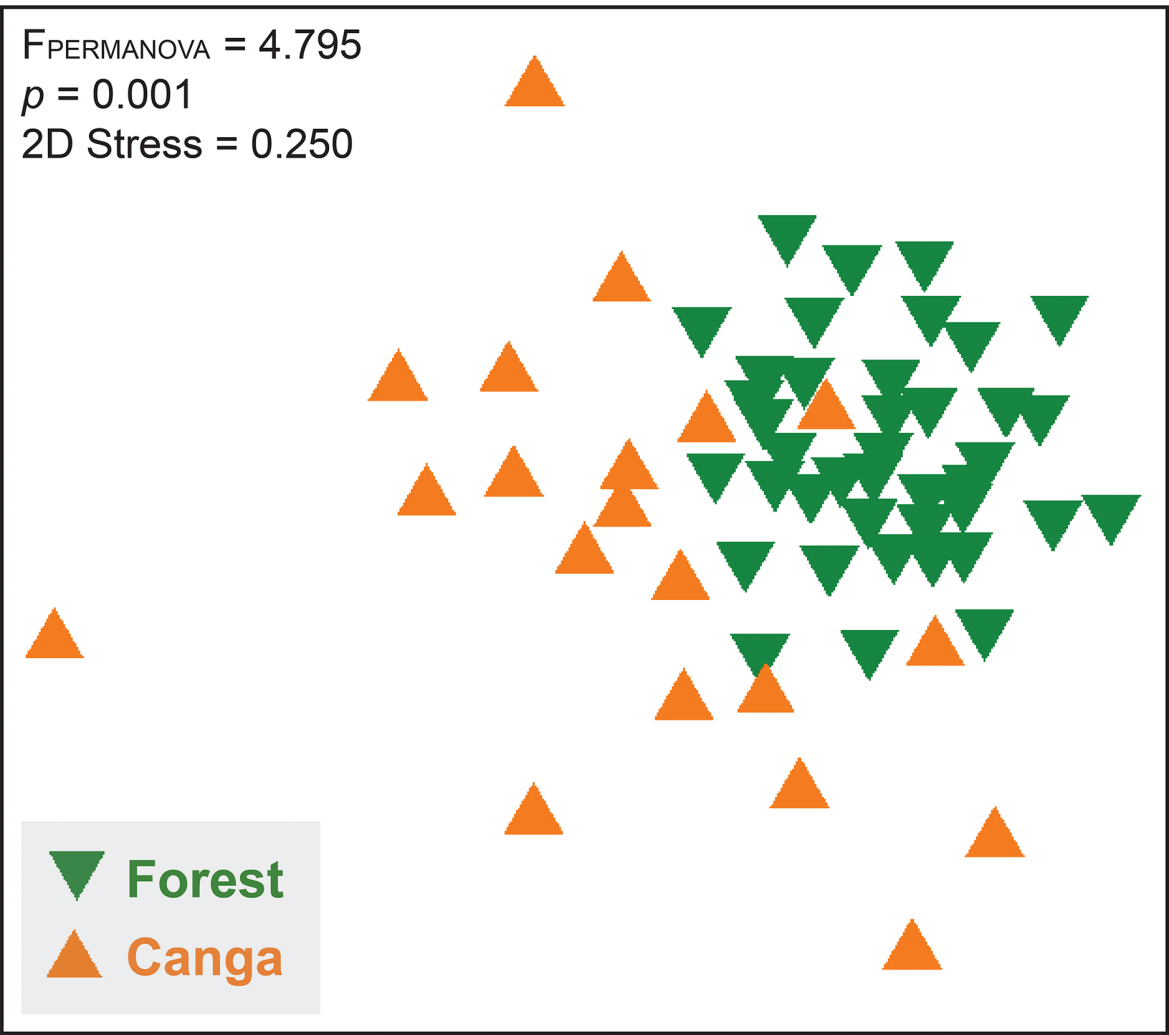
Non-metric multidimensional scaling graph (nMDS) demonstrating the differences on species composition between forest and canga transects (based on the Bray-Curtis index). PERMANOVA corroborates the faunal differences between these vegetation types according to the values of F_PERMANOVA_ and significance level (*p*).

Across all transects, we sampled 5,004 individuals belonging to 297 species (Table S2, Fig. 3). Among these, 34 species (11.45%) were shared with the adjacent caves (Fig. 4). Transects located in forested areas supported higher species richness compared to those in canga (Fig. 5A), and species composition also differed significantly between the two vegetation types (Fig. 6).

The average faunal similarity between transects and their nearest cave was 1.876% (±2.612), with a maximum similarity of 10.166% observed in a forest transect located 60 m from cave ST-0030 (Table 1). In 14 transects, we did not record any species previously found in caves (*i.e*., 0% similarity; Table 1). Forest transects harbored more species shared with the caves than canga transects (Fig. 5B). In forest areas, faunal similarity remained stable up to a distance of 77.351 m from the nearest cave, declining beyond this point (Fig. 5C). In contrast, we did not detect a significant relationship between distance and faunal similarity in canga areas (z = −1.502, *p* = 0.133).

Microhabitat type also influenced similarity patterns. Faunal assemblages associated with leaf litter were more similar to cave communities than those found under rocks and fallen tree trunks (Fig. 5D).

**Figure 7:**
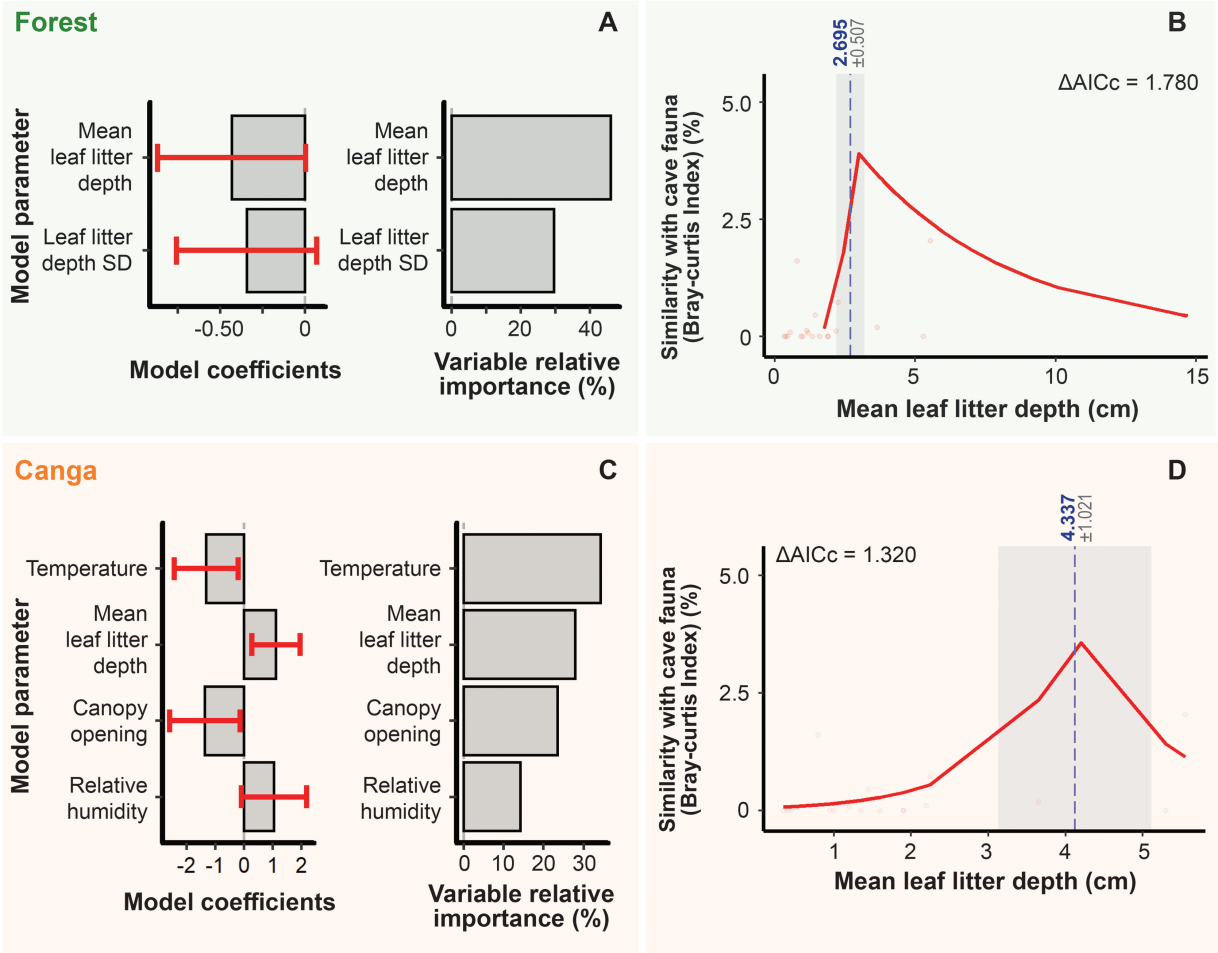
Summary of the best-fit models describing faunal similarity between caves and their surrounding environments (forest and canga). (A) Predictors influencing faunal similarity between caves and adjacent forest areas. (B) Relationship between species similarity and leaf litter depth in forest transects based on the best-fit model. (C) Predictors influencing faunal similarity between caves and adjacent canga areas. (D) Relationship between species similarity and leaf litter depth in canga transects based on the best-fit model. In panels A and C, mean coefficients (± standard error, shown in red) derived from models with Δ AICc < 2 are presented on the left, while relative importance values are displayed on the right. A predictor is considered non-significant when its standard error (red line) crosses zero. For the piecewise regressions (B and D), z, *p*, and Δ AICc values are provided. The vertical dashed blue line marks the significant breakpoint (*p* < 0.05), and the shaded gray area represents the standard error.

Among the six environmental predictors tested for forest transects, five variables were not collinear and were included in the models: temperature, relative humidity, canopy openness, and the mean and standard deviation of leaf litter depth (Table S3). Of these, only mean leaf litter depth significantly explained the similarity between forest and cave faunas, as indicated by confidence intervals excluding zero (Table 2, Fig. 7A). Similarity increased with leaf litter accumulation up to 2.695 cm, after which it progressively declined (Fig. 7B).

**Table 2:** Summary of the best-fit models describing faunal similarity between caves and epigean transects situated in forest and canga environments. For each predictor variable, the table includes the estimate, standard error (SE), z and *p* value.

Response variable	Transects location	Predictor variables	Estimate	SE	*z*	*p*
Similarity with cave fauna (Bray-Curtis index)	Forest	(Intercept)	1.763	0.456	3.869	<0.001
		Mean leaf litter depth	−0.154	0.077	−1.967	0.049
		Leaf litter depth SD	−0.143	0.261	−0.549	0.058
	Canga	(Intercept)	−1.057	0.597	1.770	0.047
		Temperature	−1.322	0.570	−2.317	0.020
		Mean leaf litter depth	1.121	0.435	2.578	0.009
		Canopy opening	−1.376	0.627	−2.194	0.028
		Relative humidity	0.549	0.584	1.794	0.073

In canga areas, the mean and standard deviation of leaf litter depth were collinear, so only the mean was retained in the models alongside temperature and canopy openness (Table S2). All three variables significantly influenced the similarity between canga and cave communities, with temperature contributing most strongly (Table 2, Fig. 7C). In contrast, relative humidity exhibited high uncertainty due to large standard errors overlapping zero and changing sign across models (Fig. 7C).

Regarding specific environmental thresholds, we observed that faunal similarity peaked at a mean leaf litter depth of approximately 4.337 cm (Fig. 7D). Higher temperatures were associated with reduced similarity between cave and canga communities (Table 2). Finally, increased canopy openness negatively affected faunal similarity, suggesting that high solar incidence limits the presence of taxa also found in caves (Table 2).

## Discussion

We found significant differences between forest and canga around the caves, both for vegetation and climatic variables and species number and composition. Such differences reflected on the faunal patterns of similarity between epigean and hypogean environments. We sampled more species common to the cave communities in forest environments, mainly close to the cavities and associated with leaf litter. In fact, we observed that leaf litter depth was determinant to the presence of troglophilic populations in both vegetation types. On the other hand, the occurrence of such species in canga areas depended on the combination of mild temperature, leaf litter accumulation and canopy thickening. Our results indicated that the maintenance of troglophilic cave communities depends on the conservation of forest areas around the caves.

### Vegetation characterization and environmental structure

Vegetation differences across the ferruginous hills are largely driven by underlying edaphic and geomorphological factors. In canga areas, soils alternate between shallow and deeper patches, reflecting active erosion processes that hinder the development of stable pedological coverage (Schaefer, 2015). These limitations restrict the establishment of larger trees, resulting in sparser canopy cover and reduced litter accumulation compared to adjacent forests. The presence of a well-structured arboreal stratum also modulates solar incidence at ground level, which is particularly relevant given the high thermal conductivity of ferruginous rocks—these substrates absorb solar radiation and efficiently transfer heat (Clauser & Huenges, 1995). Consequently, the combination of exposed rocky surfaces and low vegetative cover contributes to the elevated temperatures characteristic of canga environments.

### Diversity patterns and similarity with cave communities

Species richness and community composition responded to these environmental gradients. Previous studies have shown that vegetation type strongly influences the distribution of soil arthropods in ferruginous landscapes (*e.g*., Oliveira et al., 2019), a pattern that we now extend to broader invertebrate assemblages. The distribution of soil fauna is known to depend on physical, chemical, and ecological habitat attributes, such as temperature, moisture, vegetative cover, and prey availability (David & Handa, 2010; Beng, Corlett & Tomlinson, 2018; Basset et al., 2015). Forested habitats, with their higher resource availability and more stable microclimates, offer refugia that buffer organisms from desiccation and hydric stress—factors known to structure soil macroinvertebrate communities (De Smedt et al., 2018; Thorat & Nath, 2018; Woon et al., 2019).

The similarity between forest leaf litter and cave fauna likely results from overlapping environmental features. Like subterranean habitats, the leaf litter layer stabilizes temperature regimes, reduces solar radiation, and insulates the soil (Kostel-Hughes, Young & Wehr, 2005). This layer also modifies light quality and intensity (Schimpf & Danz, 1999), creating conditions of low light, high relative humidity, and thermal stability—features that define subterranean systems. As such, caves and forest litter may function as reciprocal habitat extensions for certain species and under certain conditions. The low similarity observed between caves and many epigean transects suggests, however, that other habitats—such as shallow subterranean habitats not sampled in this study—likely serve as important intermediaries. These subsurface habitats form a network of microvoids gradually decoupled from the surface, supporting unique communities that can act as ecotones between epigean and subterranean faunas (Gers, 1998; Mammola, Goodacre & Isaia, 2017; Ferreira, Oliveira & Silva, 2018; Oliveira & Ferreira, 2024).

The thresholds we identified for leaf litter depth may reflect hydrological and thermal constraints. Water in the litter layer exists in solid-bound, free-surface, and vapor forms, and moves through processes including interception, absorption, condensation, evaporation, and vertical transfer (Matthews, 2005; Ogée & Brunet, 2002). Coupled models of heat, water, and isotopic transport suggest that vapor flux increases with depth up to ~3 cm, and that a transition zone—an evaporative front—occurs around 4 cm under low soil moisture conditions (Haverd & Cuntz, 2010). These values align closely with the thresholds we identified for maximum similarity between cave and surface fauna. We propose that litter layers meeting these depth criteria may replicate key microhabitat conditions found in caves, facilitating the persistence of troglophilic populations. As Prous, Ferreira & Jacobi (2015) suggested, caves act as semi-permeable membranes that filter species based on environmental congruence. Where such congruence is met in surface habitats, ecological barriers may vanish, allowing species to occupy both systems seamlessly.

In canga, similarity to cave communities was observed only under specific environmental configurations resembling those of forests. We propose three non-mutually exclusive mechanisms to explain this pattern: (i) *Microhabitat availability*: Troglophilic species may colonize canga sites only when suitable microhabitats are present, particularly those offering protection from thermal and hydric stress. (ii) *Nestedness*: The canga fauna may represent a subset of adjacent forest assemblages, a pattern previously described for centipedes and millipedes in similar contexts (Oliveira et al., 2019). In this scenario, sites with higher richness tend to include species shared with caves. (iii) *Faunal exclusivity*: Canga may harbor its own specialized, xerophilic fauna, distinct from those found in subterranean systems. Indeed, we observed zero similarity in 14 transects—10 of which were located in canga—highlighting the potential for unique, endemic communities. This aligns with global evidence from ferruginous geosystems, which are often characterized by high levels of specialization and endemism (Vasconcelos & Hoffman, 2015; Buschke et al., 2012; Halse, 2018).

## Conclusion

Understanding the functional connectivity between surface and subterranean habitats is essential for biodiversity conservation. Despite their ecological uniqueness, cave communities are not entirely isolated from adjacent systems. Our results demonstrate that troglophilic species extend into forested areas surrounding ferruginous caves in the Amazon. These areas should therefore be prioritized for conservation to preserve ecological continuity across habitat types. Metrics derived from leaf litter properties may offer practical criteria to define influence zones for cave protection. In cases where forests no longer surround the caves, priority should be given to areas that approximate forest conditions—*i.e*., stable microclimates, substantial litter layers, and denser canopy cover. Future research should explore the seasonal dynamics and variability of key environmental parameters, such as litter depth, canopy openness, and temperature, to further refine our understanding of species connectivity between surface and subterranean ecosystems.

By applying these evidence-based guidelines, we believe conservation efforts targeting speleological heritage in ferruginous regions can be more effectively aligned with the dual objectives of preserving biodiversity and enabling sustainable use of the region’s exceptional mineral and biological wealth.

## Supplemental Information

10.7717/peerj.20593/supp-1 Supplemental Information 1 Comparison of environmental predictors between the two vegetation types (canga and forest) surrounding the caves. For each predictor variable, the table includes the estimate, standard error (SE), z and p value.

10.7717/peerj.20593/supp-2 Supplemental Information 2 List and number of individuals sampled in caves and epigean transects in different vegetation types I Amazon ferruginous hills. UR = Species sampled under rocks or fallen tree trunks, LL = Species sampled in the leaf litter, T = All species sampled in the epigean transect.

10.7717/peerj.20593/supp-3 Supplemental Information 3 Spearman correlation between environmental predictors measured in epigean transects in different vegetation typed in Amazon ferruginous hills. Variables were considered auto-correlated when *rho* > 0.70 (in bold). Values indicated by an asterisk were statistically significant (*p* < 0.05). The measure units of each variable are indicated in parentheses.

## Additional Information and Declarations

### Field Study Permissions

The following information was supplied relating to field study approvals (*i.e*., approving body and any reference numbers):

Permission was given by Instituto Chico Mendes de Conservação da Biodiversidade (ICMBio), N° 83/2016, regarding the protocol N° 36/2016.

### Data Availability

The following information was supplied regarding data availability:

The datasets and R scripts are available in the Supplemental Files.
