# Supplementary material for: Beneath the canopy, beneath the ground: how surface microhabitats shape cave communities
Source: PeerJ. 2026 Jan 9;14:e20593. doi: 10.7717/peerj.20593 (PMC12794640; doi:10.7717/peerj.20593)
Supplement: Supplemental Information 1 — For each predictor variable, the table includes the estimate, standard error (SE), z and p value. [file peerj-14-20593-s001.docx]

| Response variable | Predictor variables | Estimate | SE | *z* | *p* |
| --- | --- | --- | --- | --- | --- |
| Vegetation type  (Canga vs. Forest) | Temperature | -9.080 | 0.856 | -10.610 | <0.001 |
|  | Relative humidity | 0.532 | 0.051 | 10.480 | <0.001 |
|  | Canopy opening | -1.724 | 0.121 | -14.280 | <0.001 |
|  | Mean leaf litter depth | 1.080 | 0.185 | 5.831 | <0.001 |
|  | Leaf litter depth SD | 0.551 | 0.237 | 2.321 | 0.020 |
|  | Altitude | -115.28 | 22.150 | -5.205 | <0.001 |
