# Supplementary material for: Beneath the canopy, beneath the ground: how surface microhabitats shape cave communities
Source: PeerJ. 2026 Jan 9;14:e20593. doi: 10.7717/peerj.20593 (PMC12794640; doi:10.7717/peerj.20593)
Supplement: Supplemental Information 3 — Variables were considered auto-correlated when rho > 0.70 (in bold). Values indicated by an asterisk were statistically significant (p < 0.05). The measure units of each variable are indicated in parentheses. [file peerj-14-20593-s003.docx]

| **Forest** |  |  |  |  |  |  |
| --- | --- | --- | --- | --- | --- | --- |
|  | Altitude  (m. a.s.l.) | Temperature (ºC) | Humidity  (% UR) | Canopy opening (%) | Mean leaf litter depth (cm) | Leaf litter depth SD (cm) |
| Altitude (m. a.s.l.) | - | - | - | - | - | - |
| Temperature (ºC) | 0.260 | - | - | - | - | - |
| Humidity (% UR) | **-0.730*** | -0.590* | - | - | - | - |
| Canopy opening (%) | -0.190 | -0.110 | 0.003 | - | - | - |
| Mean leaf litter depth (cm) | 0.150 | 0.280* | -0.390* | -0.170 | - | - |
| Leaf litter depth SD (cm) | -0.030 | 0.250 | -0.094 | -0.330* | 0.600* | - |
| **Canga** |  |  |  |  |  |  |
|  | Altitude  (m. a.s.l.) | Temperature (ºC) | Humidity  (% UR) | Canopy opening (%) | Mean leaf litter depth (cm) | Leaf litter depth SD (cm) |
| Altitude (m. a.s.l.) | - | - | - | - | - | - |
| Temperature (ºC) | -0.520* | - | - | - | - | - |
| Humidity (% UR) | -0.240 | -0.610* | - | - | - | - |
| Canopy opening (%) | -0.059 | 0.460* | -0.490* | - | - | - |
| Mean leaf litter depth (cm) | 0.130 | -0.660* | 0.590* | -0.650* | - | - |
| Leaf litter depth SD (cm) | 0.280 | -0.570* | 0.400 | -0.590* | **0.920*** | - |
